# The effects of an interdisciplinary employment program on paid employment and mental health among persons with severe mental disorders

**DOI:** 10.1007/s00420-023-02039-7

**Published:** 2024-01-11

**Authors:** R. W. Hijdra, S. J. W. Robroek, Y. Sadigh, A. Burdorf, M. Schuring

**Affiliations:** Department of Public Health, Erasmus University Medical Center Rotterdam, PO Box 2040, 3000 Rotterdam, CA The Netherlands

**Keywords:** Severe mental disorder, Employment, Unemployment, Mental health

## Abstract

**Purpose:**

This study evaluates the effects of the interdisciplinary employment program ‘Work As Best Care (WABC)’ on employment participation and mental health of persons with severe mental disorders.

**Methods:**

WABC is a ‘work first’ employment program for unemployed persons with severe mental disorders in which employment professionals work closely together with mental health professionals. In a longitudinal non-randomized controlled study, participants of WABC (*n* = 35) are compared with participants of the control group (*n* = 37), who received regular employment support. Participants were followed for 1 year and filled out questionnaires on individual characteristics and health at baseline, after 6 and 12 months. This information was enriched with monthly register data on employment status from 2015 until 2020. Difference-in-differences analyses were performed to investigate changes in employment participation among participants of WABC and the control group. A generalized linear mixed-effects model was used to compare changes in mental health (measured on 0–100 scale) between the two groups.

**Results:**

Before WABC, employment participation was 22.0%points lower among participants of WABC compared to the control group. After starting WABC, employment participation increased with 15.3%points per year among participants of WABC, compared to 5.6%points in the control group. Among all participants of WABC, no change in mental health was found (*β* 1.0, 95% CI − 3.4; 5.5). Only female participants of WABC showed a significant change in mental health (*β* 8.0, 95% CI 2.6; 13.4).

**Conclusion:**

To enhance employment participation of persons with severe mental disorders, an interdisciplinary ‘work-first’ approach in which professionals of employment services and mental health services work in close collaboration, is of paramount importance.

**Supplementary Information:**

The online version contains supplementary material available at 10.1007/s00420-023-02039-7.

## Introduction

Severe mental disorders are attributed to be a decisive factor in loss of paid employment among 10% of all unemployed persons in the Netherlands (OECD 2014). Among persons with a severe mental disorder who are unemployed, almost 65% would like to enter paid employment (Gühne et al. 2021). However, persons with mental health problems are less likely to enter paid employment compared to persons without these problems (Yildiz et al. 2021). Persons with a severe mental disorder experience severe functional impairments in their social and societal functioning for a longer period of time (often 2 years or longer) and are in need of mental healthcare. Although severe mental disorders cannot be limited to a few diagnoses, hence in the broad definition, it not only includes psychotic disorders but also addiction and personality disorders for example (Dutch Association of Pyschiatry 2021).

Previous research has shown that paid employment can have positive effects on mental health of persons with a (severe) mental disorder (Schuring et al. 2017), reduce health care costs (Gibbons and Salkever 2019) and decrease hospitalizations (Jäckel et al. 2017). Moreover, paid employment can increase quality of life by contributing to autonomy, personal development and empowerment (Jäckel et al. 2017). Therefore, there is a clear need to enhance participation in paid employment among persons with a severe mental disorder who are able and willing to work.

Currently, few evidence-based interventions promoting employment among persons with severe mental disorders are available. Individual Placement and Support (IPS) is an evidence-based intervention that has been implemented internationally with positive effects on mental health and entering paid employment among persons with severe mental disorders (Bond et al. 2023; Frederick and VanderWeele 2019; Hellström et al. 2021; Luciano et al. 2014; Modini et al. 2016; Poulsen et al. 2022; Suijkerbuijk et al. 2017). IPS focuses on promoting employment of persons with severe mental disorders following a ‘first place then train’ method. The focus is on rapidly entering paid employment followed by training and support, depending on the needs of the client. The IPS professional is a mental health professional who supports the client in their process towards employment and builds a network of employers to create job opportunities (Drake et al. 2020). IPS participants are twice as likely to enter paid employment compared to regular employment programs. These results are seen in a wide range of countries with different unemployment levels (Modini et al. 2016). Although IPS originally started out as a re-employment program specifically for persons with severe mental disorders, it is currently expanding with positive results to other unemployed groups such as persons with common mental disorders (Probyn et al. 2021). Besides the positive employment and health effects, a systematic literature review has also shown that IPS in the short term may lead to significant welfare costs reductions which can even (partially) offset the costs of the program. The cost-effectiveness has also been shown from a healthcare perspective (Park et al. 2022).

In the Netherlands, a new employment program, Work As Best Care (WABC), has been developed. WABC builds on the same principles as the IPS program, following the ‘first place then train’ strategy, but it adds a more interdisciplinary approach. Professionals from the mental health services work in close collaboration with professionals from the employment services to promote rapid employment of persons with severe mental disorders. Professionals of the employment services have experience with the job market for unemployed persons. They utilize their broad network of local employers to identify employment opportunities. Not only does the client receive support and guidance from a mental health professional during the process towards employment but also from employment service professionals in the WABC program.

This study aims to evaluate the effects of WABC on employment participation and mental health of persons with severe mental disorders.

## Methods

### Study design and population

In this longitudinal non-randomized controlled study, the WABC group was compared with a treatment as usual group. We used a difference-in-differences approach to investigate changes in employment participation before and after implementation of WABC in comparison to the control group. A randomized controlled trial was not feasible because the referral of participants towards the WABC program or the regular employment services was controlled by the employment professionals.

From 2019 until 2020, professionals from the employment services informed persons who were enrolled in the WABC program or the regular employment services about this study. In case the client wanted to participate in the study, they were referred to the researchers. Informed consent was obtained from all participants in the study with specific consent for linking their data with information from Statistics Netherlands on employment status. Participants were followed for 12 months with three questionnaires that were administered at the start of the program, after 6 months, and at 12 months. Register data from Statistics Netherlands on employment status was used over the period 2015–2020. The Medical Ethical Committee of Erasmus MC Rotterdam declared that the Medical Research Involving Human Subjects Act does not apply to the current study (MEC-2019-0118).

**Fig. 1:**
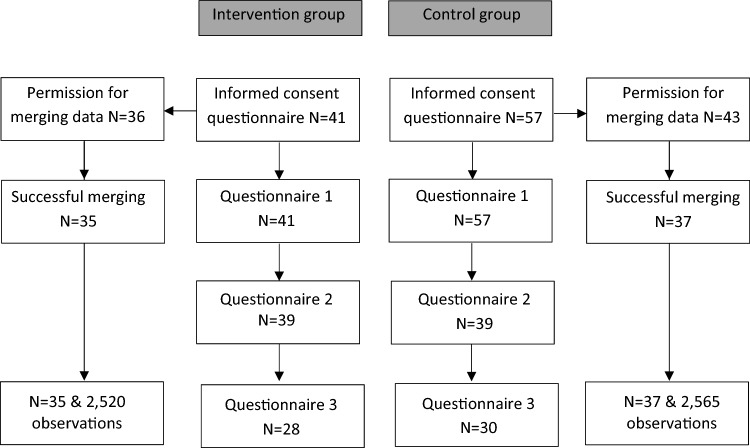
Flow chart of participants of an interdisciplinary employment program or control group

Figure 1 shows the flow of participants through the phases of the study. In total, 41 persons in the WABC group and 57 persons in the control group enrolled in the study and filled out the first questionnaire. Respectively 4 (WABC) and 14 (control) persons did not provide consent for data linkage and for 2 (WABC) and 6 (control) persons the data linkage was not successful. Finally, 35 persons of the WABC group (85%) and 37 persons of the control group (65%) were included in the merge with data from Statistics Netherlands. Enrichment of the questionnaire data with register data on employment status from 2015 up to 2020 resulted in 2520 monthly observations of 35 persons in the WABC group and 2565 monthly observations of 37 persons in the control group. Information regarding mental health was solely used from the questionnaires. All 3 questionnaires were completed by 28 persons of the WABC group and 30 persons of the control group.

### Intervention: WABC

WABC is an interdisciplinary approach in which professionals from the mental health services collaborate with professionals from the employment services. When a client with a severe mental disorder mentions to the mental health professional that he or she would like to work (more), the mental health professional contacts the employment services for the intake procedure of WABC. The mental healthcare and employment services professionals work together on a ‘work first’ approach, to find a job that is suitable for the client. Continuity of support is provided by the mental healthcare professional from the flexible assertive community treatment (FACT) team and the professional from the employment services. The FACT team consists of professionals from mental health services, social work, nurses, etc. that are specialized in treating persons with severe mental disorders (GGZ Central 2022). Severe mental disorders include, but are not limited to: psychotic disorders, bipolar disorders, personality disorders, and addiction (Dutch Association of Pyschiatry 2021). When a job opportunity is identified, the client can start a trial period at one’s new job. During the first months at the new job, the client is supported by a job coach and the professionals of WABC according to their needs. In addition, financial support is available for the employer. On average, the program lasts for a year and participants have weekly meetings with the professionals, but this can be adjusted to the individual’s needs. This includes both the process of finding the client a job as well as the support once they start a job. However, the length and frequency of the meetings can be adjusted to the client’s needs. If the participant is performing well at their new job and their mental health is stable, the meeting frequency will slowly decrease. In case a participant needed support for a longer period, this support was provided for as long as they needed. Typical features of WABC compared to the usual employment programs of the employment services are (i) continuity of care/support after entering (or losing) paid employment, (ii) job coaching after entering paid employment, and (iii) collaboration of the employment services with mental healthcare services.

### Control group: usual employment programs

The employment services support persons with social security benefits in entering paid employment. In the Dutch labor market, persons with social security benefits are required to actively look for a job. The employment services support clients in the process of searching for a suitable job by providing training to clients and by actively matching clients and employers.

### Data collection

For the WABC group, questionnaires were completed through structured interviews in person (*N* = 11) with the researcher between April 2019 and March 2020. Because of COVID-19 restrictions, the questionnaires that followed between March 2020 and December 2020 were completed through interviews by telephone with the researcher (*N* = 30). For the control group, questionnaires were sent to their home address between April 2019 and December 2020 with the possibility to schedule a phone interview. Reminders were sent twice, respectively two and four weeks after sending out the questionnaire. Questionnaire data were enriched with information from Statistics Netherlands concerning participation in paid employment.

### Participation in paid employment

Participation in paid employment was assessed with data on monthly employment status from registry data of Statistics Netherlands between 2015 and 2020. This information is derived from the Dutch tax registers. Paid employment was defined as having income from paid employment as the main source of income. With this information, employment history up to 5 years before entering this study was taken into account.

### Health

Mental health was measured by the validated 5 item Mental Health Inventory-5 (MHI-5), which measures common mental health symptoms (e.g., anxiety, depression and psychological distress) (Berwick et al. 1991; McCabe et al. 1996). Example questions of the MHI-5 are: “During the past 4 weeks, how much of the time have you felt calm and peaceful” and “During the past 4 weeks, how much of the time have you felt so down in the dumps that nothing could cheer you up?”. A 6-point answer scale was used, ranging from: “all of the time”, “most of the time”, “a good bit of the time”, “some of the time”, “a little of the time”, to “none of the time”. A sum score was then calculated and transformed to a range from 0 to 100, with a higher score indicating a better mental health (Berwick et al. 1991). The reliability of the MHI-5 has been tested with a Cronbach’s *α* of 0.84 (McCabe et al. 1996). Perceived health was measured with one validated question [‘In general, would you say your health is …’] and five answer categories “very good”, “good”, “fair”, “bad”, and “very bad”, which were clustered in three groups: (very) good, fair, and (very) bad. The reliability of the SF-12 has been tested with a Cronbach’s *α* ranging from 0.72 to 0.89 (Jenkinson and Layte 1997; Resnick and Parker 2001; Salyers et al. 2000).

### Individual characteristics

Personal information on age, sex, migration background, education, having children, and marital status were collected by the baseline questionnaire. Education was divided into three categories: low education (no education, primary education or pre-vocational training), intermediate education (vocational training), and higher education (higher secondary education and university). Marital status was used to distinguish between participants who were married and/or living with a partner from others. Migration background was divided in two categories: native Dutch (participants and parents were born in the Netherlands) and non-native Dutch (participant or at least one parent was born outside the Netherlands).

### Process evaluation

During the study, 20 semi-structured interviews and one focus group interview were conducted with professionals of WABC (*n* = 9), participants (*n* = 7) and employers (*n* = 7) to gain insights into strengths and weaknesses of the WABC strategy and barriers and facilitators for the implementation of WABC. Interviews were conducted online through Microsoft Teams or by telephone and the length ranged between 25 and 50 min. The interviews were transcribed verbatim. Afterwards, the interviews were analyzed in NVIVO (QSR International Pty Ltd 2020).

### Statistical analysis

Descriptive statistics were used to describe the WABC and control group. Logistic regression analyses were used to investigate differences in individual characteristics between the WABC and control group.

Difference-in-differences analyses were performed to investigate changes in employment participation among participants of WABC and welfare recipients receiving regular employment services (control group). First, a dichotomous variable (group) was included for the WABC (1) and control group (0). Second, a time-dependent variable was included for the time in years before the start of WABC/regular employment services (time before). An interaction term of group*time before was included in the model to test the parallel assumption referring to parallel trends in both groups before starting with WABC or regular employment services. Third, a dichotomous variable was included for the period before (0) and after (1) starting WABC or regular employment services (step change). Fourth, a time-dependent variable was included for the time in years *after* starting WABC or the regular employment services (time after). Interaction terms of group*step change and group*time after were included in the model to analyse differences between the WABC and control group in changes in employment participation in the year of and the years after starting with WABC or regular employment services. The difference-in-differences model was adjusted for social demographic characteristics (age, sex, education, migration background) and household characteristics (marital status, children). A random intercept was included in the model to account for dependency across multiple observations within individuals.

A generalized linear mixed-effects model was used to investigate changes in mental health among persons who participated in WABC or regular employment services. A variable for time in years from starting WABC or regular employment services up to December 2020, was included. Differences in baseline level and changes in time of mental health between those who participated in WABC or regular employment services were investigated with interaction terms of group*intercept and group*time. The model was adjusted for social demographic characteristics (age, sex, education, migration background). A random intercept was included in the model to account for dependency across multiple observations within individuals. Furthermore, this model was used to explore the differences between sex and age by stratifying for these variables. All analyses were performed using Stata 16.

## Results

Table 1 shows that 40.0% of the 35 participants in the WABC group and 64.9% of the 37 participants of the control group were females. A minority of the participants in the WABC group were married or living together with a partner (5.7%) and they had less often children (28.6%) compared to the control group (18.9% resp. 64.9%). The majority (68.6%) of WABC participants were aged 18–45 years, while in the control group the majority was aged 45–65 years (59.5%). In both groups, most persons had an intermediate or higher education level. Participants of the WABC group had a better perceived health compared to the control group (WABC: 48.6%, control: 73.0% with less than good perceived health), while mental health was similar among both groups. Descriptive statistics of the study population before merging with register data (Supplementary Table 1) were similar compared to the study population after merging (Table 1). Loss to follow-up analyses showed that older participants were more likely to drop-out, but other individual characteristics were not associated with loss to follow-up.

**Table 1:** Individual characteristics and (mental) health of participants of WABC group (*n* = 35) and control group (*n* = 37) whose data merging with Statistics Netherlands was successful

	WABC group *N *(%)	Control group *N* (%)
Female	14 (40.0)*	24 (64.9)
Age (years)		
18–45	24 (68.6)*	15 (40.5)
46–65	11 (31.4)*	22 (59.5)
Intermediate or high educated	24 (68.6)	30 (81.1)
Migration background	10 (28.6)	17 (45.9)
Not married or living with a partner	33 (94.3)	30 (81.1)
Children	10 (28.6)*	24 (64.9)
Less than good perceived health	17 (48.6)*	27 (73.0)

	Mean (sd)	Mean (sd)
Mental health (0–100, higher is better)	62.9 (22.8)	66.4 (24.9)

*p < 0.05 significant difference between intervention and control group

**Fig. 2:**
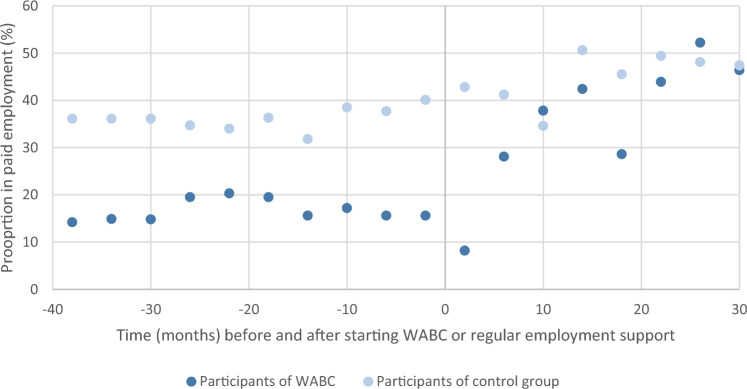
Employment participation before and after starting WABC or regular employment support in the period 2015–2020

Figure 2 shows the proportion of persons in employment in the years before and after starting WABC or regular employment services. In the years before starting WABC, persons from the WABC group were less often employed compared to the control group. After starting WABC, the proportion of persons in paid employment increased more rapidly over time compared to the control group Therefore, the difference in employment participation between both groups decreased over time.

The adjusted difference-in-differences model in Table 2 shows absolute changes of employment participation. At baseline, participation in paid employment was 22.0%points lower among participants in the WABC group, compared to the control group. In both groups, a similar yearly increase in employment participation before starting WABC or employment services was found (control group: + 2.1%points; intervention group: + 2.1%points), providing evidence that the parallel assumption of the difference-in-differences model was met. The step change in the year of starting WABC or regular employment services showed a non-significant decrease in employment participation of -5.4%points for the WABC group compared to the control group. A statistically significantly higher yearly increase in employment participation was found after starting WABC (+ 15.3%points) compared to the control group after starting regular employment services (+ 5.6%points).

**Table 2:** Difference-in-differences model for changes in employment participation in the years before and after starting WABC (*n* = 35 persons, 2520 observations) compared to changes in employment participation among welfare recipients receiving regular employment services (*n* = 37 persons, 2565 observations)

	Employment participation % points (se)			
	WABC group	Control group	Difference between groups	
			Unadjusted model	Adjusted model^
Employment participation at baseline	18.77 (3.29)	39.14 (5.32)	− 19.52 (6.15)*	− 21.99 (6.91)*
Yearly change before WABC/employment services	2.12 (0.62)*	2.06 (0.94)*	1.09 (1.21)	1.64 (1.21)
Step-change in the year of starting WABC/employment services	− 4.21 (2.82)	2.61 (3.78)	− 6.01 (4.22)	− 5.38 (4.24)
Yearly change after starting WABC/employment services	15.34 (1.75)*	5.63 (1.96)*	11.77 (2.34)*	11.87 (2.36)*

*p < 0.05 significant difference between intervention and control group

^Adjusted for age, sex, education, migration background, and household characteristics (marital status and children)

Table 3 shows that mental health did not change in the WABC group (*β* 1.0, 95% CI − 3.4; 5.5) nor in the control group (*β* − 2.1, 95% CI − 5.6; 1.5). The adjusted model shows that the difference in yearly change between the WABC group and the control group was 4.9 points per year, but this difference was not statistically significant (95% CI − 1.3; 11.2). However, among women, a significantly higher increase in mental health was found in the WABC group (*β* 8.0, 95% CI 2.6; 13.4), compared to the control group (*β* − 3.4, 95% CI − 7.1; 0.4) (Supplementary Table 2).

**Table 3:** Changes in mental health (scale 0–100) among persons who participated in WABC or regular employment services

	WABC group B (95% CI)	Control group B (95% CI)	Difference (unadjusted) B (95% CI)	Difference (adjusted)^ B (95% CI)
Estimated baseline (*N* = 41, WABC; *n* = 57, control)	64.37 (58.04–70.629)	67.02 (61.60–72.44)	2.65 (− 5.68–10.99)	− 1.17 (− 10.43–8.10)
Change in time (*n* = 39, WABC; *n* = 39, control)	1.04 (− 3.42 5.50)	− 2.05 (− 5.58–1.47)	3.09 (− 2.60–8.77) (*p* = 0.287)	4.94 (− 1.34–11.22) (*p* = 0.123)

*p < 0.05 significant difference between intervention and control group

^Adjusted for age, sex, education, migration background, and household characteristics (marital status and children)

The process evaluation showed that persons were positive about WABC. Professionals from the employment services and mental health services mentioned that the interdisciplinary collaboration improved employment trajectories of participants and they appreciated working together. However, they also mentioned that they needed more guidance on how to approach the multidisciplinary aspect of WABC and utilize their specific expertise. Employers appreciated the personal contact and guidance they received from the employment services, and the ability to hire an employee with a trial period. Participants mentioned that they valued the personal contact with the professionals of WABC as well as the guidance from the job coach. However, both employers as well as participants mentioned that sometimes the professionals were busy and hard to reach.

## Discussion

The interdisciplinary work-first employment program (WABC) for persons with severe disorders had a positive influence on employment participation, but an effect on mental health was only found among female participants. With a stronger increase in employment participation among participants of WABC compared to the control group, the difference in employment participation between both groups was strongly reduced.

Participants of WABC showed a stronger increase in employment participation compared to the control group. Within two years, the large difference in employment participation between the WABC and control group was almost diminished. The sustained increase in employment participation in the years after starting WABC suggests that the participants were able to maintain employment after ending the WABC program. These promising results need to be compared to other studies evaluating employment programs based on the same principles. A systematic literature review showed that participants of IPS were 1.6 times more likely to enter paid employment compared to persons who followed a regular employment program (Frederick and VanderWeele 2019). In addition, a study from Switzerland evaluating an IPS program found that after 6 years, 45% of the participants were still in paid employment (Pichler et al. 2021). It is difficult to draw conclusions on whether WABC is more effective than IPS, because these studies differ in study design, study population and contextual factors. The results of the WABC program, with a combined approach of the employment services and the mental health services, are promising, but further research is needed to corroborate the findings.

Only among female participants of WABC, a change in mental health was found. This could be due to potential differences in mental disorders and recovery between men and women (Boyd et al. 2015; Schön 2010). Among all persons who started WABC, no improvement in mental health was observed. This is in line with results from a systematic review and meta-analysis performed by van Rijn et al. They reported no evidence for an improved mental health among persons with a severe mental disorder who entered a re-employment program (van Rijn et al. 2016). A study performed specifically in the Netherlands on IPS also showed no change in mental health in both the IPS and the control group before and after starting the program. However, they did find an improvement in mental health among persons who entered employment compared to persons who did not enter employment (Carlier et al. 2018). In the current study, analyses of the difference in mental health between persons who did and did not enter paid employment could not be performed because of a lack of power. Furthermore, methodological or contextual reasons should be considered to interpret the lack of significant improvements in mental health. The measurement instruments may for example not be able to capture changes in (severe) mental health disorders of the participants. The MHI-5 assessed symptoms for common depressive disorders and anxiety (Berwick et al. 1991), and does not provide information on other mental disorders. Last but not least, the small number of participants may have caused problems with the power of the analysis. Further research is recommended to investigate the effect of (different types of) employment on mental health among persons with severe mental disorders.

Although all participants of WABC had a severe mental disorder, the prevalence of severe mental disorders in the control group was unknown. Both groups had similar scores on the MHI-5 scale, but this scale only measures common mental disorders, such as depression and anxiety, and is not able to capture the presence of other severe mental disorders (Berwick et al. 1991). Previous research has shown that unemployed persons have a decreased self-reported health (Böckerman and Ilmakunnas 2009). Mental disorders are quite common among persons who are unemployed, with a prevalence of over 18% (Pinto-Meza et al. 2013). In addition, there was a difference in age-distribution between the WABC and control group. This may have had an effect on the type and severity of mental disorders since the onset of most mental disorders occurs during adolescence or early adulthood (Solmi et al. 2022). Because it was unknown whether participants of the control group had a severe mental disorder, a difference-in-differences approach was used. This method takes into account differences in unobserved characteristics, by focusing on changes in time within groups instead of differences between groups. However, the trends in the outcome measures need to be similar in both groups before starting the intervention. The results showed that the parallel assumption of the difference-in-differences model was met.

The strength of this study was the combination of register data and self-reported questionnaire data as well as the use of an analytical strategy from econometrics for causal inference in a non-randomized controlled study. Because the questionnaire data were enriched with data from Statistics Netherlands, detailed information on employment status was available without having to burden participants or professionals involved with WABC. This increased the number of observations and the power of the study. Furthermore, with the difference-in-differences approach it was possible to evaluate the influence of the WABC program on employment participation by using longitudinal observational data. Lastly, the data from this study was nationally representative for persons with severe mental disorders as we recruited participants from organizations with legal responsibility for care.

However, this study also had several limitations. First, the number of participants was low, which can partly be explained by the strict selection criteria of WABC and the COVID-19 pandemic happening halfway through this study. This is especially the case for the stratified analysis for sex in supplementary Table 2. The COVID-19 restrictions of the Dutch government caused difficulties for the collaboration and client contact because of working from home mitigation measures. This also resulted in changes in jobs (e.g., working from home, limited number of persons in shops or restaurants, etc.) and the labor market. There was a large demand for employees at the time of this study. Second, some participants were already in paid employment before starting the WABC program or regular employment services. These persons were likely only working a few hours a week or were involved in sheltered employment and preferred to work more hours in regular employment. Third, it was unknown whether the control group had severe mental disorders. This problem was addressed by the difference-in-differences method—focusing on changes in health or employment participation in time, taking into account time-constant differences between persons. Fourth, because register data was used, there was almost no loss to follow-up on employment status. There was a loss to follow-up with the questionnaires. If this was due to persons with worse mental health not being able to continue participating in this research, a general increase in mental health would be expected. However, this increase was not shown in the results. Thus, the reasons behind the loss to follow-up in the questionnaires remains unknown. Lastly, there was a difference in data collection between the WABC and control group, which may influence the results. Previous research showed that participants often report a better health in telephone interviews compared to paper questionnaires (Feveile et al. 2007; Lungenhausen et al. 2007). This may have reduced the differences in self-reported (mental) health between the WABC group and the control group.

Professionals of employment services are often reluctant to guide persons with a severe mental disorder towards paid employment. In practice, persons with severe mental disorders receive little to no employment support from employment services (de Winter et al. 2020). Professionals from employment services are not trained to guide persons with mental health problems towards employment, but by working in close collaboration with mental health services they have the opportunities to actively guide persons with mental health problems towards employment (Rijksoverheid 2021). With the support of the WABC program, persons with mental disorders who were not expected to participate in the labor market, successfully entered paid employment. Having a severe mental disorder should not be an exclusion criterion for receiving support towards paid employment. The interdisciplinary approach of professionals of the employment services and mental health services is seen as a critical factor in supporting the full work capacity of persons with (severe) mental disorders.

To enhance employment participation of persons with severe mental disorders as well as common mental disorders, an interdisciplinary ‘work-first’ approach in which professionals of employment services and mental health services work in close collaboration, is of paramount importance. Policymakers and funding organizations should encourage and accommodate such programs and collaborations. To expand the positive effects of WABC on paid employment, the program could also be evaluated among persons with common mental disorders.

### Supplementary Information

Below is the link to the electronic supplementary material.

Supplementary file1 (DOCX 13 kb)

## Data Availability

The data that support the findings of this study are available from Statistics Netherlands, but restrictions apply to the availability of these data, which were used under license for the current study and so are not publicly available. The data are available from Statistics Netherlands.
