# Supplementary material for: The effects of an interdisciplinary employment program on paid employment and mental health among persons with severe mental disorders
Source: Int Arch Occup Environ Health. 2024 Jan 11;97(3):253–62. doi: 10.1007/s00420-023-02039-7 (PMC10944804; doi:10.1007/s00420-023-02039-7)
Supplement: Supplementary file 1 — Supplementary file1 (DOCX 13 kb) [file 420_2023_2039_MOESM1_ESM.docx]

Supplementary table 1 Individual characteristics and (mental) health of participants of WABC (n=41) and welfare recipients who received regular employment services (n=57) based on questionnaire information

|  |  |  |
| --- | --- | --- |
|  | WABC group  N(%) | Control group  N(%) |
| Female | 17 (41.5) | 38 (66.7) |
| Age (years)  18-45  46-65 | 28 (70.0)*  12 (30.0)* | 21 (36.8)  36 (63.2) |
| Intermediate or high educated | 29 (70.8) | 38 (67.9) |
| Migration background | 15 (26.6) | 27 (48.1) |
| Not married or living with a partner | 39 (95.1) | 45 (78.9) |
| Children | 12 (29.3)* | 37 (64.9) |
| Less than good perceived health | 19 (46.3) | 24 (60.8) |
|  | Mean (sd) | Mean (sd) |
| Mental health (0-100, higher is better) | 63.7 (21.6) | 66.9 (22.3) |

*p<0.05 significant difference between intervention and control group

Supplementary table 2 Changes in mental health among males (n=42; 96 observations) and females (n=54; 120 observations) who participated in WABC or regular employment services

|  | WABC group  B (95%CI) | Control group  B (95%CI) | Difference (unadjusted)  B (95%CI) | Difference  (adjusted)^  B (95%CI) |
| --- | --- | --- | --- | --- |
| Estimated baseline | | | | |
| Males | 66.34 (58.26 - 74.41) | 62.08 (53.27 - 70.89) | 4.26 (-7.69 - 16.21) | 9.07 (-3.20 - 21.34) |
| Females | 60.07 (50.20 - 69.94) | 69.60 (62.86 - 76.34) | -9.53 (-21.48 - 2.42) | -10.96 (-22.99 - 1.06) |
| Change in time (per year) | | | | |
| Males | -1.59 (-9.67 - 6.49) | 1.09 (-5.22 - 7.40) | -2.68 (-12.93 - 7.57) | -3.49 (-13.66 - 6.69) |
| Females | 8.02 (2.63 - 13.41) | -3.36 (-7.14 - 0.42) | 11.38 (4.79 - 17.96)* | 13.09 (6.30 - 19.88)* |

^Adjusted for age, sex, education, migration background and household characteristics (marital status and children).*p<0.05 significant difference between intervention and control group
